# Adipose-derived stem cells repair radiation-induced chronic lung injury via inhibiting TGF-β1/Smad 3 signaling pathway

**DOI:** 10.1515/med-2023-0850

**Published:** 2023-11-09

**Authors:** Xin Huang, Wei Sun, Bin Nie, Juan-juan Li, Fei Jing, Xiao-li Zhou, Xin-ye Ni, Xin-chu Ni

**Affiliations:** Department of Radiotherapy, The Affiliated Changzhou Second People’s Hospital of Nanjing Medical University, Changzhou Second People’s Hospital, Changzhou Medical Center, Nanjing Medical University, Changzhou, Jiangsu, 213000, China; Department of Pathology, The Affiliated Changzhou Second People’s Hospital of Nanjing Medical University, Changzhou Second People’s Hospital, Changzhou Medical Center, Nanjing Medical University, Changzhou, Jiangsu, 213000, China; Department of Radiotherapy, The Affiliated Changzhou Second People’s Hospital of Nanjing Medical University, Changzhou Second People’s Hospital, Changzhou Medical Center, Nanjing Medical University, No. 68, Gehuzhonglu Road, Wujin District, Changzhou, Jiangsu, 213000, China

**Keywords:** adipose-derived stem cells, radiation-induced lung injury, fibroplasia, TGF-β1/Smad 3 signaling pathway

## Abstract

To investigate the effect of adipose-derived stem cells (ASCs) transplantation on radiation-induced lung injury (RILI), Sprague-Dawley rats were divided into phosphate-buffered saline (PBS) group, ASCs group, Radiation + PBS group, and Radiation + ASCs group. Radiation + PBS and Radiation + ASCs groups received single dose of 30 Gy X-ray radiation to the right chest. The Radiation + PBS group received 1 mL PBS suspension and Radiation + ASCs group received 1 mL PBS suspension containing 1 × 10^7^ CM-Dil-labeled ASCs. The right lung tissue was collected on Days 30, 90, and 180 after radiation. Hematoxylin–eosin and Masson staining were performed to observe the pathological changes and collagen fiber content in the lung tissue. Immunohistochemistry (IHC) and western blot (WB) were used to detect levels of fibrotic markers collagen I (Collal), fibronectin (FN), as well as transforming growth factor-β1 (TGF-β1), p-Smad 3, and Smad 3. Compared with the non-radiation groups, the radiation groups showed lymphocyte infiltration on Day 30 after irradiation and thickened incomplete alveolar walls, collagen deposition, and fibroplasia on Days 90 and 180. ASCs relieved these changes on Day 180 (Masson staining, *P* = 0.0022). Compared with Radiation + PBS group, on Day 180 after irradiation, the Radiation + ASCs group showed that ASCs could significantly decrease the expressions of fibrosis markers Collal (IHC: *P* = 0.0022; WB: *P* = 0.0087) and FN (IHC: *P* = 0.0152; WB: *P* = 0.026) and inhibit the expressions of TGF-β1 (IHC: *P* = 0.026; WB: *P* = 0.0152) and p-Smad 3 (IHC: *P* = 0.0043; WB: *P* = 0.0087) in radiation-induced injured lung tissue. These indicated that ASCs could relieve RILI by inhibiting TGF-β1/Smad 3 signaling pathway.

## 1 Introduction

Radiotherapy plays a fundamental role in the treatment and management of thoracic cancers. Nevertheless, radiotherapy-related toxicities, such as radiation-induced lung injury (RILI), are pivotal dose-limiting factors that directly affect the prognosis and life quality of patients [1]. Pneumonia and fibrosis are common in lung tissues exposed to irradiation [2]. A high-dose radiotherapy for thoracic cancer resulted in RILI in more than 40% of patients and even death in a minority [3]. Unfortunately, there are few safe and effective strategies for treating RILI.

RILI is usually divided into an early-stage radiation-induced pneumonitis and late-stage radiation-induced pulmonary fibrosis (RIPF) [4]. RIPF is an incurable heterogeneous disease characterized by scar formation leading to progressive and irreversible destruction of pulmonary structures, which ultimately resulted in pulmonary dysfunction, disruption of air exchange, and even death from respiratory failure [4–6]. To date, the underlying mechanism of RILI is still not well defined. Transforming growth factor-β1 (TGF-β1), as a pleiotropic cytokine generated by activated immune cells and alveolar type I or II epithelial cells, has been reported to be crucial in the pulmonary fibrosis as it can promote the activation of myofibroblasts, proliferation of fibroblasts, and deposition of extracellular matrix (ECM) [7–9]. Furthermore, TGF-β1/Smad 3 signaling pathway has been reported to undertake a pivotal role in the differentiation of fibroblast to myofibroblast, sustained myofibroblast activation, and aberrant ECM deposition [10,11]. On this basis, it is reasonable to speculate that the inhibition of TGF-β1/Smad 3 signaling pathway may hinder the progression of RILI.

Mesenchymal stem cells (MSCs) have been used to repair tissue damage including lung injury [12]. Among MSCs, adipose tissue-derived MSCs (ASCs) have been used in tissue injury repairment as its convenient acquisition, rapid proliferation *in vitro*, multi-directional differentiation even after long-term culture, and low immunogenicity [13]. At present, there are many studies of ASCs on TGFβ1/Smad 3 signaling pathway in some tissues such as skin [14], muscle [15], and liver [16]. However, in radiation-induced injured lung tissue, the intervention of ASCs on TGFβ1/Smad 3 signaling pathway has not been reported. Here, we aimed to investigate the roles of ASCs in RILI of Sprague-Dawley (SD) rats. Furthermore, we tried to reveal the underlying mechanism by which ASCs exert their roles.

## 2 Materials and methods

### 2.1 Animals

One hundred and thirty adult female SD rats (200 ± 20 g) of specific pathogen free grade were purchased from Shanghai Institute of Family Planning Sciences (certificate No.: 20180006030261; License No.: SCXK 2018-0006; Shanghai, China). Animals were fed with a standard diet and kept in a clean animal room.


**Ethical statement:** The animal experimental protocol was approved by the Ethical Committee of Nanjing Medical University (approval number: IACUC-2107055).

### 2.2 Isolation and culture of ASCs

Thirty rats were used for the isolation of ASCs. Briefly, rats were anesthetized by intraperitoneal injection of 1% pentobarbital (40 mg/kg). Then the subcutaneous adipose tissue was excised from the groin. Upon removal of blood vessels, the tissues were cut into pieces and placed in a centrifuge tube. Afterward, the mixture was digested with 40 mL I-type collagenase solution (0.075%) for 60 min in shaking water bath at 37°C. The mixture was transferred to a 50 mL centrifuge tube and centrifuged at 1,500 rpm for 10 min. The pellet was suspended with Dulbecco’s Modified Eagle’s Medium and fetal bovine serum (10%), followed by incubation in a 100 mL culture dish at 37°C for 24 h in an incubator with atmosphere of 5% CO_2_ and a saturated humidity. Finally, the primary (P0) ASCs were obtained after removing floating impurity cells rinsed with phosphate-buffered saline (PBS). The culture medium was changed twice a week. Cells were passaged at a ratio of 1:3 upon reaching 80% confluence.

### 2.3 Identification and labeling of ASCs

The identification of ASCs was performed based on flow cytometry for the labeling of the surface markers of passage3 (P3) ASCs. The surface was labeled with rhodamine B isothiocyanate (RBITC)-conjugated anti-CD45 rabbit monoclonal antibody, fluorescein isothiocyanate (FITC)-conjugated anti-CD31 rabbit monoclonal antibody, and FITC-conjugated anti-CD90 mouse monoclonal antibody (Abcam Inc., Cambridge, MA). A nonspecific RBITC/FITC-conjugated IgG (Abcam Inc., Cambridge, MA) was used as an isotype control to assess the background fluorescence. Analysis was done by flow cytometer using the CellQuest Pro software. ASCs (P2) were digested, centrifuged, resuspended in PBS, and labeled with chlormethylbenzamido-1,1′-dioctadecyl-3,3,3′,3′-tetramethyl indocarbocyanine (CM-DiI) (Invitrogen, USA) according to the manufacturer’s instructions. The labeled ASCs were placed in a dark environment for subsequent culture.

### 2.4 Experimental design

Seventy-two SD rats were randomly divided into (i) PBS group (*n* = 18), injected with 1 mL PBS via tail vein; (ii) ASCs group (*n* = 18), injected with 1 mL PBS containing 1 × 10^7^ allogeneic CM-Dil-labeled ASCs via tail vein; (iii) Radiation + PBS group (*n* = 18), injected with 1 mL PBS via tail vein at 2 h after a single radiation dose of 30 Gy to right chest; and (iv) Radiation + ASCs group (*n* = 18), injected with 1 mL PBS containing 1 × 10^7^ allogeneic CM-Dil-labeled ASCs via tail vein at 2 h after a single radiation dose of 30 Gy to right chest. Animals were anesthetized by intraperitoneal injection of 1% pentobarbital (40 mg/kg) before radiation. Source-skin distance radiation was carried out using 6 MeV electrons generated by an Elekta infinity linear accelerator (Elekta, Sweden) at 3 cm depth in supine position using a constant dose rate of 600 cGy/min. General behaviors and conditions of the rats were observed daily. Then six rats randomly selected from each group were sacrificed on Days 30, 90, and 180, respectively. Subsequently, the right lung was obtained, and the tissues were stored for subsequent analysis.

### 2.5 Homing analysis

Twenty-eight rats were divided into the following groups, including (i) ASCs group, injected 1 × 10^7^ allogeneic CM-Dil-labeled ASCs via tail vein and (ii) Radiation + ASCs group, injected 1 × 10^7^ allogeneic CM-Dil-labeled ASCs via tail vein at 2 h after a single radiation dose of 30 Gy to right chest. One rat was sacrificed in each group per day until 14 days after the ASCs injection to obtain the right lung. The homing of ASCs was observed under a fluorescence microscope based on the prepared frozen sections.

### 2.6 Histological analysis

The freshly harvested tissues were fixed with 10% formaldehyde for 24 h, dehydrated with alcohol, and then embedded in paraffin wax. To reflect the general structure and alveolitis status of lung tissue, the slides were performed hematoxylin–eosin (HE) staining as previously published [17]. Masson staining was performed to mainly reflect the fibrosis in lung tissue, and five fields were randomly selected as previously described [18]. Fibrosis degree was quantified by the percentage of positive collagen fiber area to total area using Image J. Immunohistochemistry (IHC) was performed as described elsewhere [19]. Briefly, the tissue sections were deparaffinized and rehydrated. Endogenous peroxidase activity was blocked with 3% H_2_O_2_ for 10 min. Then slides were placed in a citrate buffer solution and heated in a microwave for 10 min for antigen retrieval, followed by blocking in normal serum to reduce the background. Afterward, the tissue sections were washed three times in PBS solution for 10 min and incubated overnight at 4°C with primary rabbit monoclonal antibodies against collagen I (Collal), fibronectin (FN), TGF-β1, Smad 3, and p-Smad 3 (1: 50; Abcam Inc., Cambridge, MA). Slides were then washed with PBS solution, followed by incubation with horseradish peroxidase-conjugated secondary antibody for 1 h at room temperature. Slides were washed again in PBS solution and developed using 3,3′-diaminobenzidine working solution, and the termination of the reaction was determined using microscope. The positive alveolar epithelial cells were observed under a microscope. Integral optical density (IOD) analysis was performed based on images of each section captured from five random fields by semi-quantitative scoring system using Image Pro Plus 6.0.

### 2.7 Western blot (WB) analysis

Protein extraction was performed according to the instructions of the kit (Keygen Bio Tech, Nanjing, China) after the homogenization of snap-frozen lung tissues. Protein samples were separated using sodium dodecyl sulfate polyacrylamide gel electrophoresis (Keygen Bio Tech, Nanjing, China) and transferred onto a polyvinylidene fluoride membrane. Then the membranes were blocked with 5% skimmed milk for 90 min and incubated overnight at 4°C with primary rabbit monoclonal antibodies against Colla1, FN, TGF-β1, p-Smad 3, and Smad 3 (Abcam Inc., Cambridge, MA). Then the membrane was washed in Tris-buffered saline and Tween (TBST) and incubated with the corresponding secondary antibody for 90 min at room temperature. After washing three times in TBST, images were acquired with an electro-chemiluminescence kit (Keygen Bio Tech, Nanjing, China). Signal intensities of bands were quantified using Image J as described in the previous study [20]. Membranes probed with GAPDH were used as internal reference.

### 2.8 Statistical analysis

Statistical analysis was performed using SPSS 16.0 software. All measurement data were expressed as mean ± standard deviation (SD). The comparison between groups was performed by Wilcoxon Mann–Whitney *U* test. Hypothesis testing was two-tailed. A *P*-value of less than 0.05 was considered to be statistically significant.

## 3 Results

### 3.1 Morphology and specific antigen expression of ASCs

**Figure 1:**
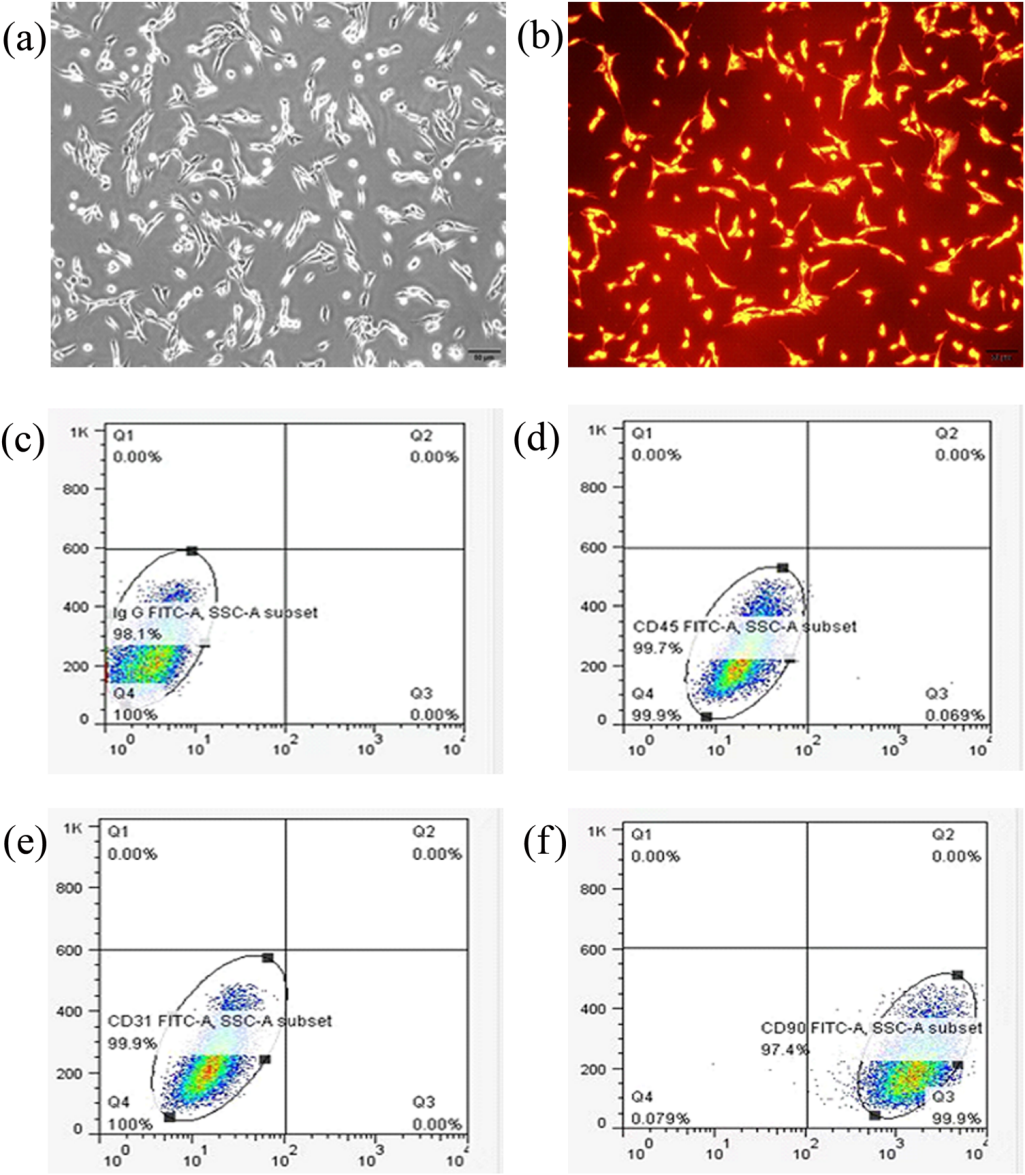
Morphology of ASCs and specific antigen expression in ASCs surface. (a) Microscopic images of ASCs in passage three (P3). (b) Fluorescence microscopic images of CM-Dil-labeled ASCs. (c) IgG isotype control group. (d) There was no expression of hematopoietic stem cell-specific antigen CD45 in ASCs. (e) No expression of endothelial cell-specific antigen CD31 was found in ASCs. (f) There was ASC-specific antigen CD90 in ASCs.

ASCs were spindle-shaped fibroblast-like adherent cells, with plump and centered nuclei (Figure 1a). The cell membrane and cytoplasm of ASCs labeled with CM-Dil showed uniform red fluorescence roughly consistent with the cell shape under a fluorescence microscope at 553 nm wavelength. The nuclei were not stained (Figure 1b). Compared with IgG isotype control (Figure 1c), both hematopoietic stem cell-specific antigen CD45 and endothelial cell-specific antigen CD31 showed extremely low or even no expression in ASCs (CD45: 0.07%; CD31: 0%; Figure 1d and e). ASCs-specific antigen CD90 was highly expressed with a positive rate of 97.4% (Figure 1f).

### 3.2 Tracking of ASCs based on CM-Dil labeling

**Figure 2:**
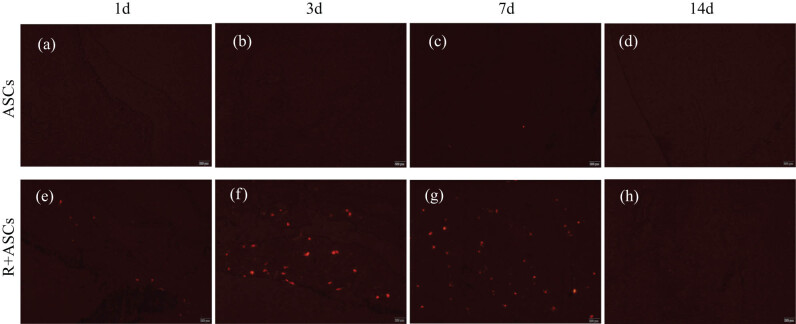
Fluorescence microscope pictures of frozen lung tissue sections after tail vein injection of CM-Dil-labeled ASCs in SD rats. Bar = 200 μm. In the ASCs group, there were no CM-Dil-labeled ASCs on Day 1 (a) and Day 3 (b) after ASCs transplantation. (c) An extremely small number of CM-Dil-labeled ASCs were observed on Day 7. (d) No CM-Dil-labeled ASCs were observed on Day 14. (e) In the Radiation + ASCs group, ASCs were sporadically distributed on Day 1. There were adequate CM-Dil-labeled ASCs on Day 3 (f) and Day 7 (g). (h) Only a spot of CM-Dil-labeled ASCs were observed on Day 14.

For the tracking of ASCs in rats, no CM-Dil-labeled ASCs were observed on Days 1 and 3 after ASCs transplantation in ASCs group (Figure 2a and b). There was an extremely small number of CM-Dil-labeled ASCs in ASCs group 7 days after cellular transplantation (Figure 2c). No CM-Dil-labeled ASCs were observed on Day 14 (Figure 2d). In the irradiation group, the CM-Dil-labeled ASCs were sporadically distributed in lung tissues since the day of transplantation (Figure 2e). On Days 3 and 7, there were adequate CM-Dil-labeled ASCs in the lung tissues in Radiation + ASCs group (Figure 2f and g), and only a spot of CM-Dil-labeled ASCs were observed on Day 14 (Figure 2h). On Days 30, 90, and 180, no CM-Dil-labeled ASCs were observed in both the groups. This showed that ASCs can home to injured lung tissues. However, with the decay of fluorescence ASCs could not be seen in injured lung tissue on Days 30, 90, and 180.

### 3.3 Histopathological changes of lung tissues

**Figure 3:**
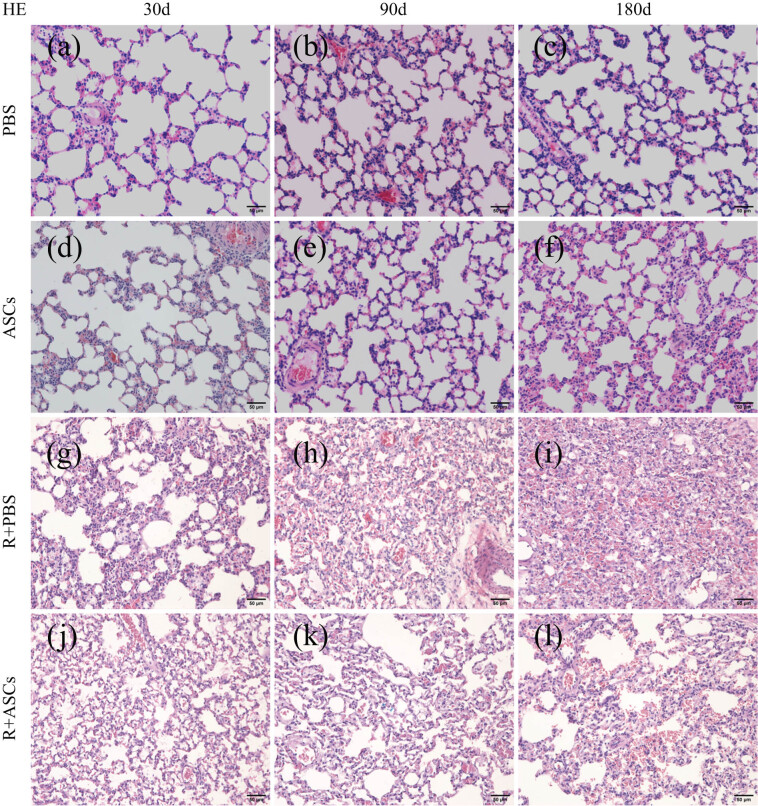
HE-staining images (Bar = 50 μm) of lung tissues in PBS, ASCs, Radiation + PBS, and Radiation + ASCs groups on Days 30, 90, and 180 of ASCs injection. (a–f) No obvious exudation and fibroplasia were found in lung tissues in PBS and ASCs groups. (g and j) Acute inflammation including congestion, edema, and inflammatory cell infiltration were seen on Day 30 after irradiation in Radiation + PBS and Radiation + ASCs groups. (h and k) Chronic inflammatory changes featured by lymphocyte infiltration and fibroplasia were seen on Day 90 after irradiation in Radiation + PBS and Radiation + ASCs groups. (i) In Radiation + PBS group, alveolar wall was incomplete and obviously thickened, together with the presence of massive lymphocytes and obvious fibroplasia. (l) These changes were significantly attenuated in Radiation + ASCs group.

**Figure 4:**
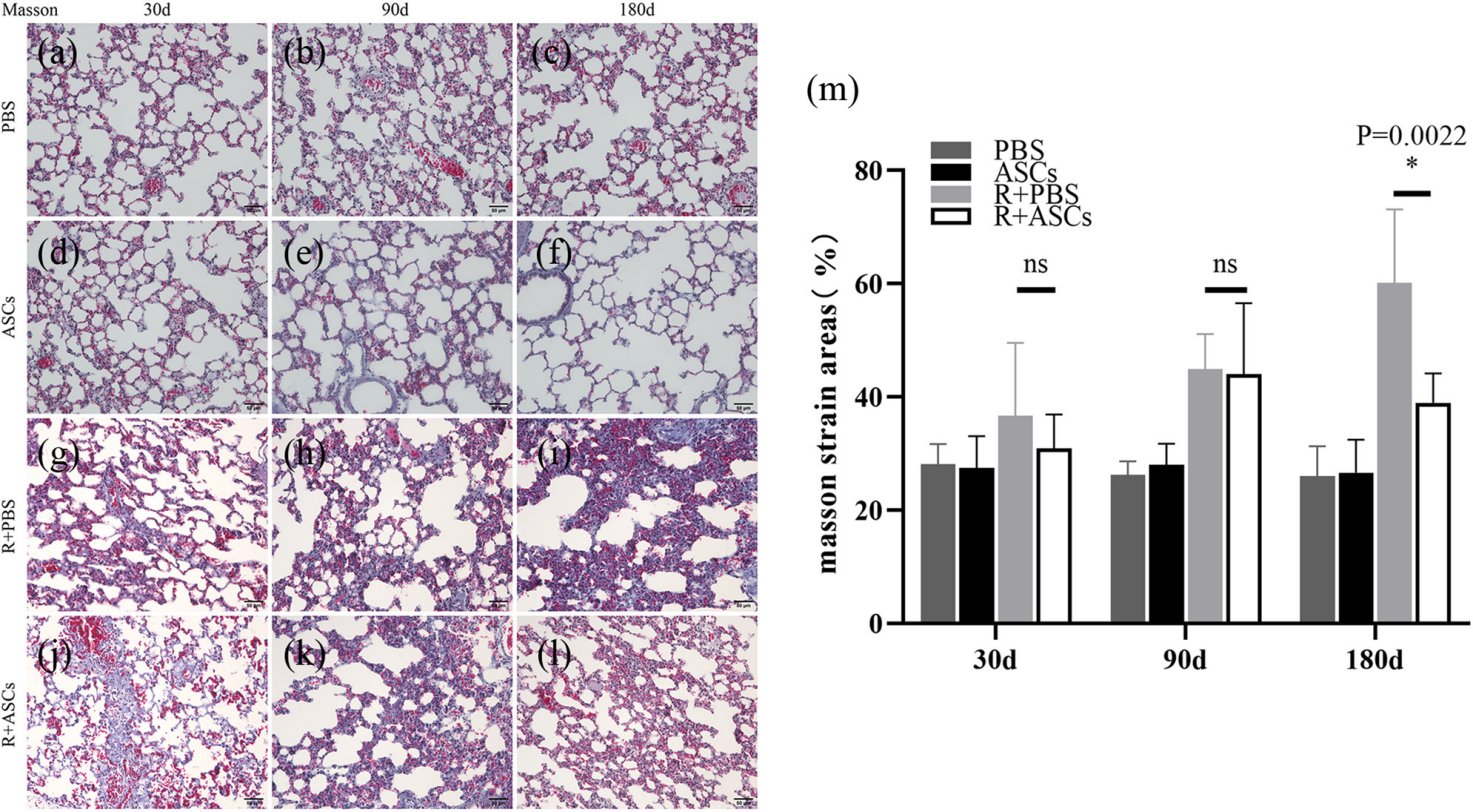
Masson staining images (Bar = 50 μm) of lung tissues in PBS, ASCs, Radiation + PBS, and Radiation + ASCs groups on Days 30, 90 and 180 of ASCs injection. (a–f) There were no obvious collagen fibers in lung tissues in PBS and ASCs groups. (g, h, j, and k) On Days 30 and 90, there was no significant difference in collagen fiber area in the Radiation + ASCs group compared with that in Radiation + PBS group. (i and l) On Day 180, the collagen fiber area was significantly decreased in the Radiation + ASCs group compared with that in Radiation + PBS group. (m) On Day 180, ASCs relieved fibrosis degree in chronic lung injury induced by irradiation (*P =* 0.0022, Wilcoxon Mann–Whitney *U* test). For each group, *n* = 6. *P < 0.05, ns means no statistical difference.

HE staining indicated that the alveolar structure of lung tissues in PBS group and ASCs group was intact, with no obvious exudation and fibroplasia on Days 30, 90, and 180, respectively (Figure 3a–f). Masson staining also showed no obvious formation of collagen fibers in PBS group and ASCs group (Figure 4a–f). In Radiation + PBS group, acute inflammation including congestion, edema, and inflammatory cell infiltration was seen in lung tissues on Day 30 after irradiation (Figure 3g). No significant differences of these changes were observed in Radiation + ASCs group (Figure 3j). Slight Masson staining was seen on Day 30 after irradiation in two groups (Figure 4g and j). In addition, chronic inflammatory changes featured by lymphocyte infiltration in lung tissues were seen on Day 90, along with fibroplasia in pulmonary tissues in Radiation + PBS group (Figure 3h). No significant difference of the change was observed in Radiation + ASCs group (Figure 3k). Consistently, Masson staining of Radiation + PBS and Radiation + ASCs groups showed scattered collagen fibers, with no significant difference in the percentage of collagen fiber-positive area to total area (Figure 4h, k, and m). On Day 180, the alveolar wall was incomplete and obviously thickened after irradiation together with the presence of massive lymphocytes and obvious fibroplasia in Radiation + PBS group (Figure 3i); however, these conditions were significantly attenuated in Radiation + ASCs group (Figure 3l). Masson staining also showed that on Day 180, compared with that in Radiation + PBS group, there was significant decrease in collagen fiber area in the Radiation + ASCs group (Figure 4i, l, and m, *P* = 0.0022). This showed that ASCs can inhibit fibroplasia and attenuate chronic lung injury induced by irradiation.

### 3.4 ASCs inhibited fibroplasia

**Figure 5:**
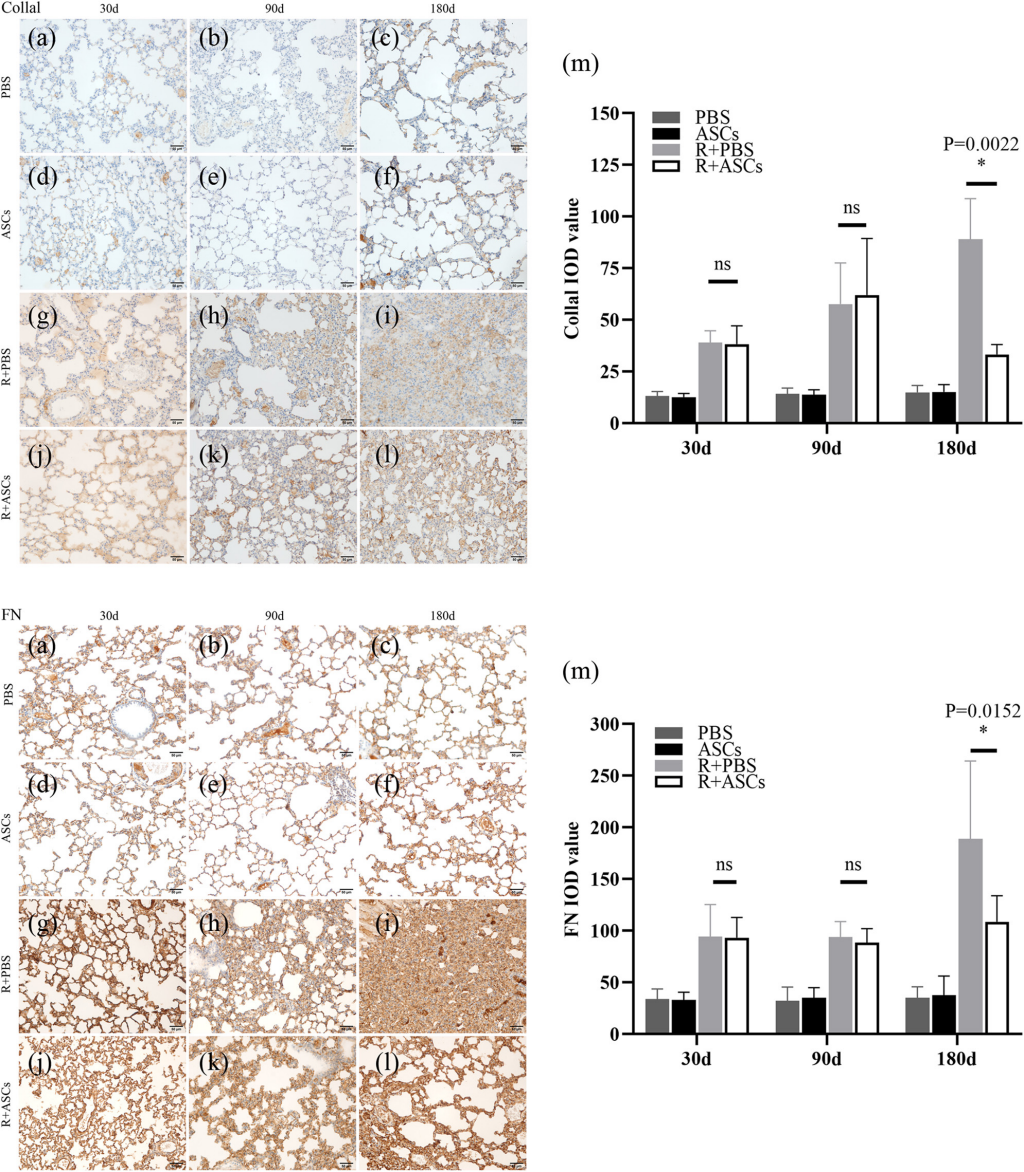
IHC images (bar = 50 μm) and IOD analysis of Collal and FN in PBS, ASCs, Radiation + PBS, and Radiation + ASCs groups on Days 30, 90, and 180 after irradiation. (a–f) No expressions of Collal and FN were found in lung tissues in PBS and ASCs groups. (g, h, j, and k) On Days 30 and 90, there were no significant differences in expressions of Collal and FN between the Radiation + ASCs group and Radiation + PBS group. (i and l) On Day 180, the expressions of Collal and FN were significantly decreased in the Radiation + ASCs group compared with that in Radiation + PBS group. (m) On Day 180, ASCs decreased fibrosis degree in chronic lung injury induced by irradiation (Collal: *P =* 0.0022, FN: *P =* 0.0152, Wilcoxon Mann–Whitney *U* test). For each group, *n* = 6. *P < 0.05, ns means no statistical difference.

**Figure 6:**
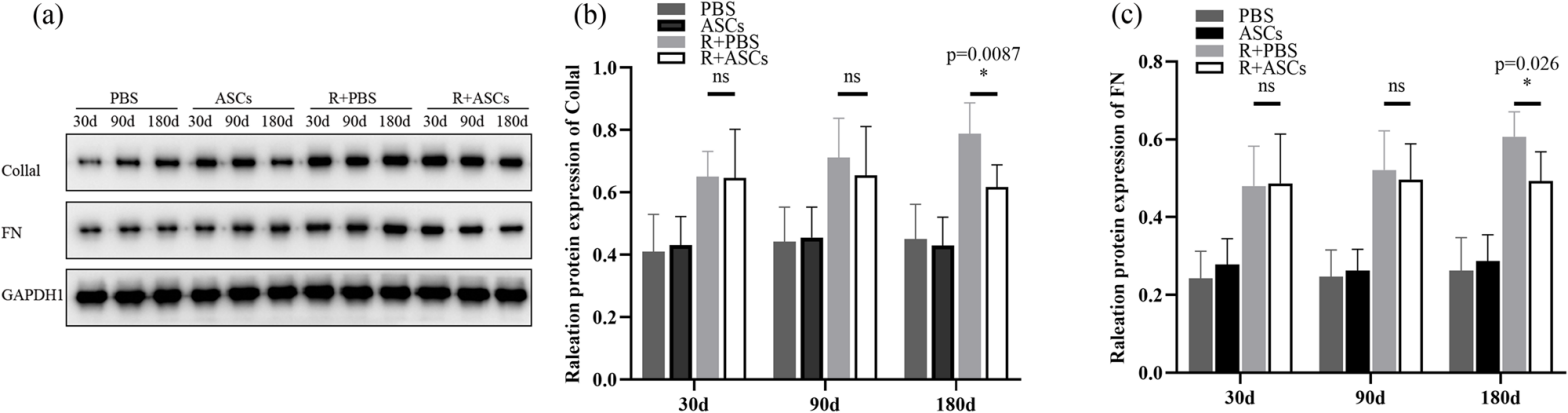
WB and quantitative analysis of Collal and FN in PBS, ASCs, Radiation + PBS, and Radiation + ASCs groups on Days 30, 90, and 180 after irradiation. (a) Representative bands of western blotting. (b and c) On Day 180, the protein expressions of Collal (*P =* 0.0087, Wilcoxon Mann–Whitney *U* test) and FN (*P =* 0.026, Wilcoxon Mann–Whitney *U* test) were significantly decreased in the Radiation + ASCs group compared with that in Radiation + PBS group. The density values of blots were normalized to the internal control GAPDH1. Data were expressed as mean ± standard deviation. For each group, *n* = 6. *P < 0.05, ns means no statistical difference.

IHC and WB analysis results together indicated that no statistical differences in the expressions of Collal and FN were found in PBS and ASCs groups on Days 30, 90, and 180. The expressions of Collal and FN between Radiation + PBS and Radiation + ASCs groups showed no statistically significant differences on Days 30 and 90. On Day 180, the expressions of Collal (IHC: *P* = 0.0022; WB: *P* = 0.0087) and FN (IHC: *P =* 0.0152; WB: *P* = 0.026) were significantly down-regulated in Radiation + ASCs group compared with that in Radiation + PBS group (Figures 5 and 6). This showed that ASCs could inhibit fibroplasia in chronic lung injury induced by irradiation.

### 3.5 ASCs repaired RILI via inhibiting TGF-β1/Smad 3 signaling pathway

**Figure 7:**
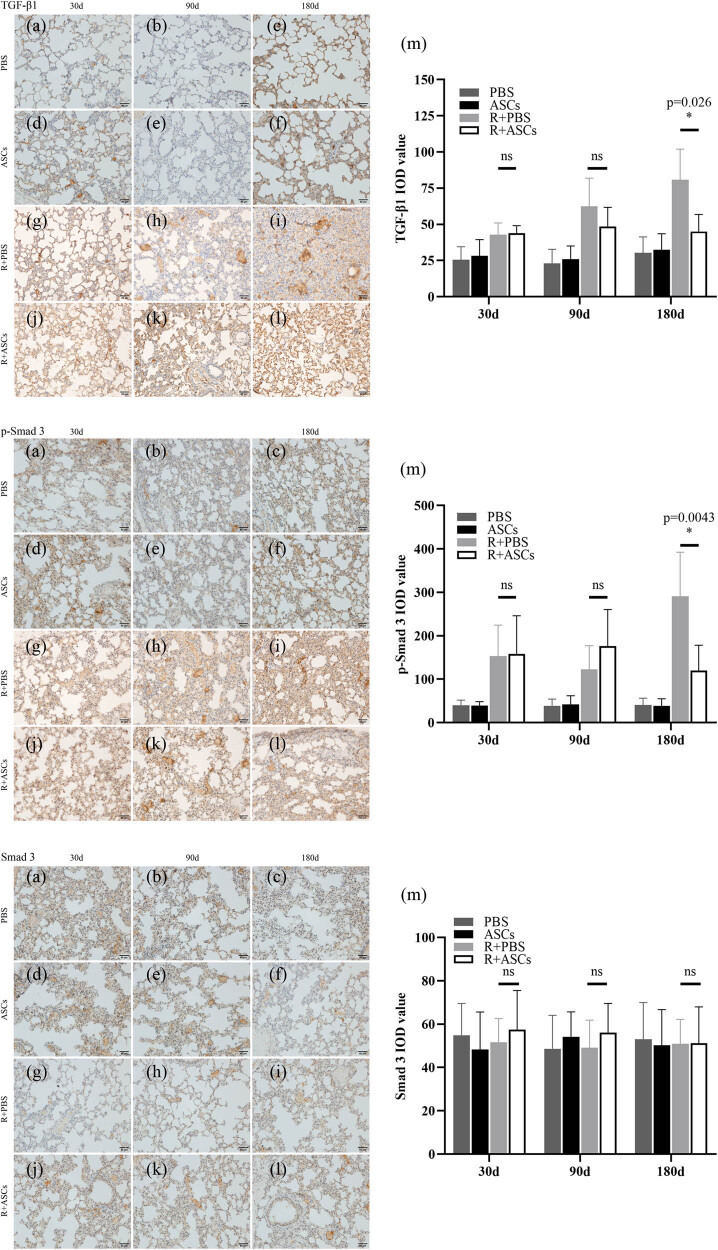
IHC images (bar = 50 μm) and IOD analysis of TGF-β1, p-Smad 3, and Smad 3 in PBS, ASCs, Radiation + PBS, and Radiation + ASCs groups on Days 30, 90, and 180 after irradiation. (a–f) There were no expressions of TGF-β1, p-Smad 3, and Smad 3 in lung tissues in PBS and ASCs groups. (g, h, j, and k) On Days 30 and 90, no significant differences in the expressions of TGF-β1, p-Smad 3, and Smad 3 were found between the Radiation + ASCs group and Radiation + PBS group. (i and l) On Day 180, the expressions of TGF-β1and p-Smad 3 were significantly decreased in the Radiation + ASCs group than that in the Radiation + PBS group. (m) On Day 180, ASCs decreased the levels of TGF-β1 (*P =* 0.026, Wilcoxon Mann–Whitney *U* test) and p-Smad 3 (*P =* 0.0043, Wilcoxon Mann–Whitney *U* test) in chronic lung injury induced by irradiation. For each group, *n* = 6. *P < 0.05, ns means no statistical difference.

**Figure 8:**
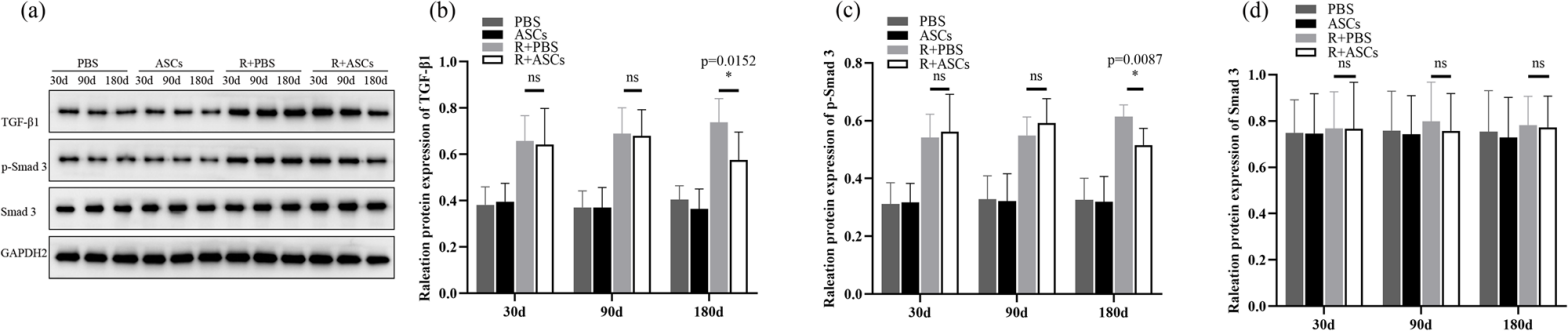
WB and quantitative analysis of TGF-β1, p-Smad 3, and Smad 3 in PBS, ASCs, Radiation + PBS, and Radiation + ASCs groups on Days 30, 90, and 180 after irradiation. (a) Representative bands of western blotting. (b–d) On Day 180, compared with Radiation + PBS group, ASCs decreased the protein expressions of TGF-β1 (*P* = 0.0152, Wilcoxon Mann–Whitney *U* test) and p-Smad 3 (*P* = 0.0087, Wilcoxon Mann–Whitney *U* test). The density values of blots were normalized to the internal control GAPDH2. Data were expressed as mean ± standard deviation. For each group, *n* = 6. *P < 0.05, ns means no statistical difference.

Expressions of TGF-β1, p-Smad 3, and Smad 3 in PBS and ASCs groups showed no statistically significant differences at all timepoints. Compared with Radiation + PBS group, the levels of TGF-β1 and p-Smad 3 showed no statistical difference in Radiation + ASCs group on Days 30 and 90 after irradiation. On Day 180 after irradiation, the levels of TGF-β1 (IHC: *P =* 0.026; WB: *P* = 0.0152) and p-Smad 3 (IHC: *P =* 0.0043; WB: *P* = 0.0087) were significantly down-regulated in Radiation + ASCs group than that in Radiation + PBS group. This implied that ASCs can inhibit TGF-β1/Smad 3 signaling pathway in chronic lung injury induced by irradiation. No difference of Smad 3 expression was observed between Radiation + PBS and Radiation + ASCs groups at all timepoints (Figures 7 and 8).

## 4 Discussion

The results in this study revealed that ASCs could inhibit fibroplasia, attenuate RILI, and inhibit the expressions of TGF-β1 and p-Smad 3 proteins. To our knowledge, this is the first study revealing that ASC could repair RILI by inhibiting the TGF-β1/Smad 3 signaling pathway. Recently, numerous studies have focused on the treatment of RILI, a common post-radiotherapy complication in patients with thoracic cancers [21–23]. In a clinical study on the characteristics and dosimetry of RIPF, 48 enrolled patients received 44–65 Gy irradiation, among whom 39 cases finally developed RIPF, with a dose-dependent manner [24]. RILI animal models have usually been successfully established using a dose of 15–20 Gy irradiation on whole chest or right chest [25–28]. For those studies involving higher-dose irradiation, Vujaskovic et al. utilized 28 Gy irradiation on right hemithorax, and diffuse interstitial fibrosis was observed in the irradiated lung tissue after 6 months of irradiation [29]. Kwock et al. adopted an acute (30 Gy) or fractionated (5 × 6 Gy) dose to one hemithorax of rats, and pulmonary vascular injury was observed after 4–6 weeks of irradiation [30]. In this study, a higher-dose (30 Gy) irradiation was given to the right chest of SD rats to induce RILI. The results showed that the RILI rat model could be successfully established using a higher-dose irradiation. Given that patients often receive higher-dose irradiation, the RILI rat model established by higher-dose irradiation in our study is of great clinical significance.

Homing of ASCs to the injured lung tissues is crucial for RILI repairment. Based on real-time PCR, Ortiz et al. reported that ASCs injected via tail vein could home to the injured lung tissue through the circulation [31]. Through bioluminescence imaging and real-time PCR, Baer et al. found that Atm-deficient mice still showed a strong bioluminescence signal on Day 9 after injection, and the bioluminescence signal was decreased to undetectable level on Day 14. Interestingly, based on real-time PCR, a positive Luc signal in the lung parenchyma still could be detected on Day 50 after injection of ASCs isolated from luciferase transgenic mice (mASCs), which indicated that ASCs could reside in the injured lung tissue for a long time and participate in injury repairment [32]. Jiang et al. reported that the level of ASCs in the RILI lung tissues reached a peak after 72 h of injection by *in vivo* optical imaging technology and observation of frozen sections. However, ASCs could not be detected on Day 14 after injection [25]. In this study, we explored the homing of ASCs by tail vein injection of CM-Dil-labeled ASCs. The results showed that CM-Dil-labeled ASCs were densely distributed in lung tissues on Days 3–7 after injection in Radiation + ASCs group. Only a spot of CM-DiL-labeled ASCs were observed on Day 14. On Days 30, 90, and 180, no CM-DiL-labeled ASCs were observed. Consistently, our findings suggested that ASCs could home to injured lung tissues, which indicated that CM-Dil tracking was a pivotal tool for observing homing of ASCs in injured lung tissues.

The pathogenesis of RILI is complex, involving multiple factors and cells. Cytokines are highly related to the onset of RILI. Various cytokines regulate the pathogenesis and progression of RILI by forming a cytokine network through autocrine, paracrine, and mutual interaction [33,34]. Among them, TGF-β1 was a representative of pro-inflammatory cytokine and chemokine for radiation-induced injury [35,36]. In the presence of irradiation exposure, TGF-β1 generated by immune cells, alveolar cells, and fibroblasts could regulate fibroblast proliferation in lung tissue via the activation of multiple signaling cascades mediated by the Smad protein family [37–39]. It has been reported that fibroblasts could express Collal and FN, with the capacity to differentiate into myofibrillar cells, while the extra-cellular expression of Collal and FN could lead to alveolar sclerosis and decrease of pulmonary function [40]. TGF-β1/Smad 3 signaling pathway could participate in pulmonary fibrosis of RILI by the expression of Collal and FN [41,42]. In a previous study on stem cell therapy for RILI, irradiation to the whole chest led to pulmonary fibrosis in mice, manifested as up-regulation of TGF-β1, which were effectively reversed by ASCs transplantation [43]. In addition, Hao et al. [44] transplanted human umbilical cord mesenchymal stem cells (UC-MSCs) to beagle dogs with localized irradiation to the right lower lung, which showed that UC-MSCs could alleviate RILI in the chronic phase about 180 days after transplantation. Moreover, Jiang et al. reported that ASCs could reduce the significant up-regulation of TGF-β1 and Collal, and alleviate RILI in rats received 15 Gy irradiation to the right lung on Day 28 [25]. Our results showed that on Day 180, the alveolar wall was incomplete and obviously thickened after irradiation with the presence of obvious fibroplasia in Radiation + PBS group; however, these conditions were significantly attenuated in Radiation + ASCs group. In our study, the levels of fibrosis markers FN and Collal protein in Radiation + ASCs group were significantly lower than those in Radiation + PBS group on Day 180 after the ASCs transplantation, indicating that ASCs could inhibit fibroplasia and alleviate RILI. Furthermore, the levels of TGF-β1 and p-Smad 3 protein in Radiation + ASCs group were significantly lower than that in Radiation + PBS group on Day 180 after the transplantation of ASCs. Therefore, the mechanism by which ASCs attenuated RILI may be inhibition of the activation of the TGF-β1/Smad 3 signaling pathway.

This study has some limitations. First, the sample size is small; therefore, the conclusion still needs to be confirmed by studies with large sample size. Besides, RILI is a complex process involving different signaling pathways at different stages [45]. Except for TGF-β1/Smad 3 signaling pathway, whether ASCS can act on other signaling pathways to repair RILI was not revealed in this study. In our next work, we will explore other possible signaling pathways and molecular mechanism of ASCs in the process of repairing RILI through *in vivo* and *in vitro* experiments based on a large sample size. We look forward to providing more theoretical basis for the clinical treatment of RILI.

## 5 Conclusion

In summary, ASCs could inhibit fibroplasia and attenuate chronic lung injury induced by irradiation. Inhibiting TGF-β1/Smad 3 signaling pathway was considered to involve in the attenuation of RILI by ASCs. In the future, ASCs transplantation may be a clinical effective strategy for the treatment of chronic lung injury induced by irradiation.
